# Protection and Response of a Tertiary Hospital in Shenzhen, China to the COVID-19 Outbreak: The Practice of the Comprehensive Response Mode

**DOI:** 10.1017/dmp.2021.218

**Published:** 2021-07-12

**Authors:** Benling Hu, Le Yang, Chan Wei, Min Luo

**Affiliations:** 1 Breast Surgery, The University of Hong Kong–Shenzhen Hospital, Shenzhen, China; 2 Emergency Department, Shenzhen University General Hospital, Shenzhen, China

**Keywords:** novel coronavirus pneumonia, COVID-19, general hospital, prevention and control measures

## Abstract

**Objective:**

The aim of this study was to evaluate the management mode for the prevention and control of coronavirus disease 2019 (COVID-19) transmission used at a general hospital in Shenzhen, China, with the aim to maintain the normal operation of the hospital.

**Methods:**

From January 2, 2020, to April 23, 2020, Hong Kong–Shenzhen Hospital, a tertiary hospital in Shenzhen, has operated a special response protocol named comprehensive pandemic prevention and control model, which mainly includes 6 aspects: (1) human resource management; (2) equipment management; (3) logistics management; (4) cleaning, disinfection, and process reengineering; (5) environment layout; (6) and training and assessment. The detail of every aspect was described, and its efficiency was evaluated.

**Results:**

A total of 198,802 patients were received. Of those, 10,821 were hospitalized; 26,767 were received by the emergency department and fever clinics; 288 patients were admitted for observation with fever; and 324 were admitted as suspected cases for isolation. Under the protocol of comprehensive pandemic prevention and control model, no case of hospital-acquired infection with COVID-19 occurred among the inpatients or staff.

**Conclusion:**

The present comprehensive response model may be useful in large public health emergencies to ensure appropriate management and protect the health and life of individuals.

In December 2019, an outbreak of a novel coronavirus was reported in Wuhan, China. On February 11, the World Health Organization (WHO) formally named the novel coronavirus pneumonia coronavirus disease 2019 (COVID-19). By May 5, 2020, there were 84,407 confirmed cases and 4643 deaths in China. At present, significant progress has been made in the prevention and control of COVID-19 in China. Efforts are focused on the prevention and control of COVID-19 transmission to resume work and production internally and prevent the influx of imported cases from abroad. According to China’s Law on the Prevention and Control of Infectious Diseases, local governments should designate hospitals with the necessary resources and ability to undertake the task of treating infectious diseases or set up appropriate facilities for this purpose. The Shenzhen Third People’s Hospital is the only designated hospital for the diagnosis and treatment of patients with COVID-19 in Shenzhen, China (according to the requirements of the Municipal Health and Health Commission). In addition, The University of Hong Kong–Shenzhen Hospital is a designated hospital for the treatment of patients suspected of COVID-19 and foreign patients in Shenzhen. Currently, there is no specialist department or experience in the centralized treatment of patients with infectious diseases. Therefore, it is necessary to construct a new prevention and control mode under the existing conditions. The elements of this mode and the actions to be taken at each step of the process are described in detail below.

## Methods

### Human Resource Management

The University of Hong Kong–Shenzhen Hospital has 2000 beds, including 1577 inpatient beds, 80 intensive care unit (ICU) beds, and 68 observation beds. Annually, the hospital serves approximately 60,000 inpatients, 1.44 million outpatients, and 720,000 emergency patients. During the COVID-19 pandemic, early detection, reporting, diagnosis, isolation, and treatment of cases are required to control the transmission of the disease, reduce the patient infection rate, and prevent the infection of medical staff. Arrangements for the screening and reception of patients, in triage, fever clinic, isolation ward, and the inpatient department should be made to control the source of infection, disrupt the chain of transmission, and protect the susceptible population.

### Establishment of a 3-Level Triage System

**Figure 1.**
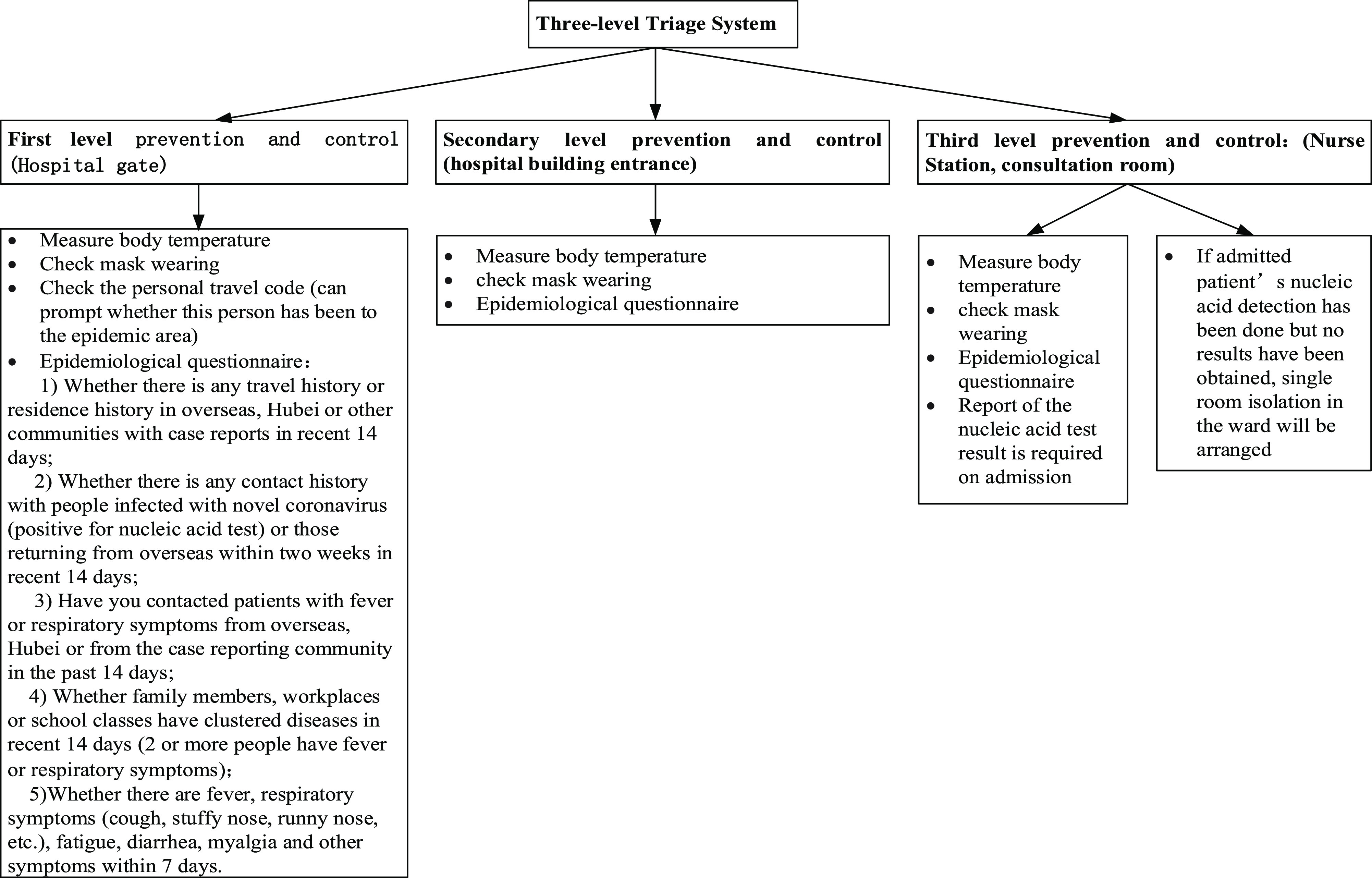
Three-level triage system.

Under the 3-level prevention and control system (Figure 1), a joint prevention and control mechanism should be formed in which all staff are responsible for the prevention and control of COVID-19 transmission.^1^

#### First Level of Prevention and Control

The entrance and exit of pedestrians and vehicles should be reduced. All hospital visitors must wear masks, be subjected to temperature monitoring, and complete the COVID-19 epidemiological questionnaire in electronic or paper form. Those with warning signs/symptoms should be referred to the outpatient or emergency triage site for further triage.

#### Second Level of Prevention and Control

Preliminary screening of patients who arrive at the outpatient department or emergency department for the detection of warning signs/symptoms should be performed for all patients, visitors, and staff who enter the inpatient building.

#### Third Level of Prevention and Control

For patients entering the consultation area, emergency department, and inpatient department, the 2-dimensional code, triage result, epidemiological history, and COVID-19 epidemiological history form (signed by the patient and nurse); the body temperature of patients should also be measured as needed. A negative nucleic acid test, conducted within 7 d, is required for both patients and accompanying persons on admission. Those without a test should be admitted to a single room for isolation.

If the epidemiological history and presence of clinical symptoms are confirmed by the outpatient or emergency triage points, the patient shall be taken to the fever clinic by a designated person.

Patients with an epidemiological history of COVID-19 shall be confirmed at the outpatient or emergency triage points and brought to the emergency for investigation and screening. Patients aged <50 y, without epidemiological history, and presenting only with fever (frontal/axillary temperature ≥37.3°C, ear temperature ≥37.3°C) should be triaged to the fever consultation room in the emergency department.

If any hospital visitor other than the patient meets any of the epidemiological history criteria and presents clinical symptoms, the outpatient, emergency department, and inpatient entrance personnel shall directly inform the designated person to guide them to the fever clinic. If the visitor reports an epidemiological history without clinical symptoms within 14 d, they should not be allowed to enter the hospital area. For those presenting with only clinical symptoms or abnormal body temperature, emergency department consultation is recommended.

### Policy of Patient Admission

#### Patients Suspected of COVID-19

**Figure 2.**
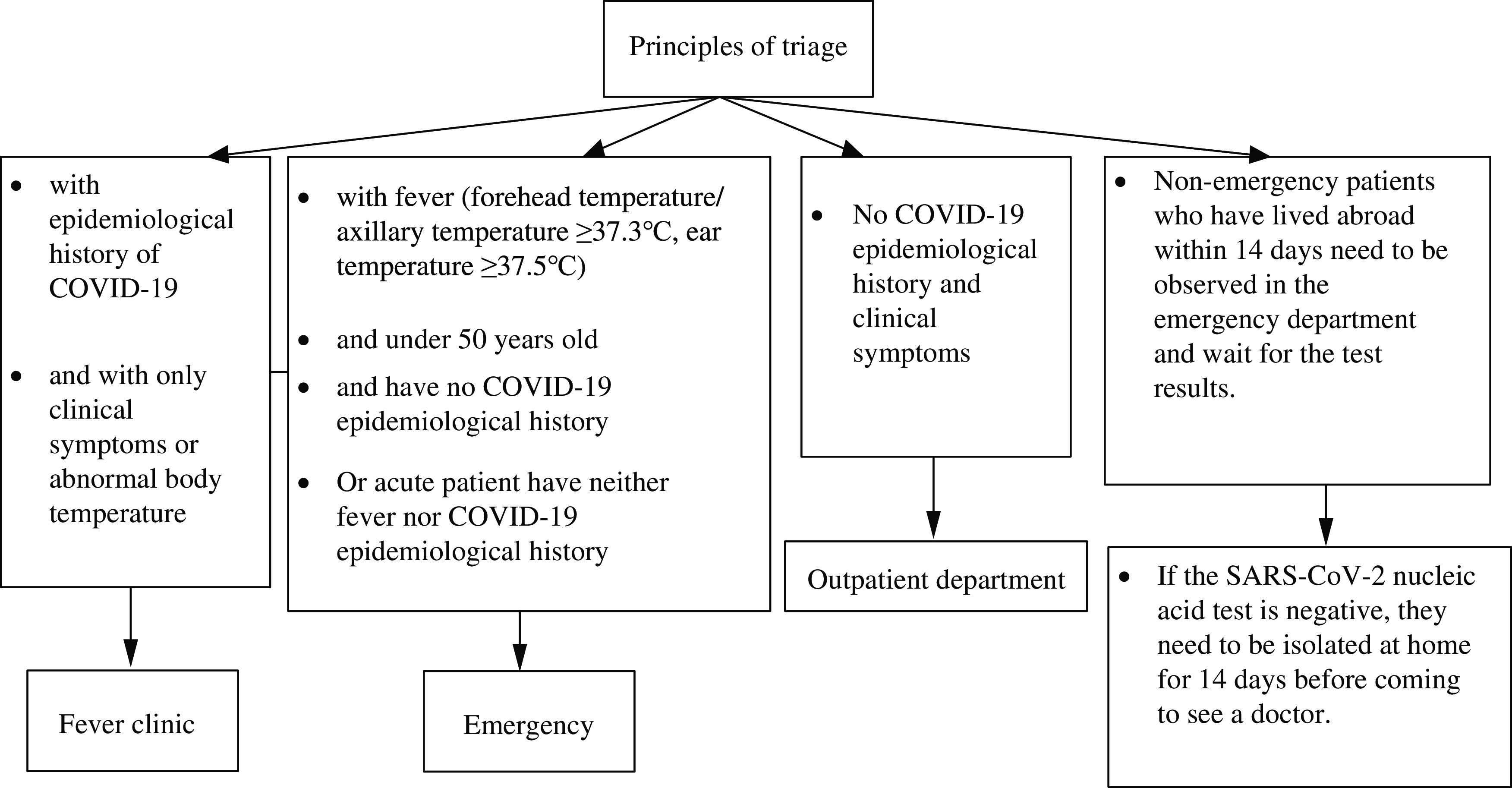
Principles of triage.

As shown in Figure 2, there are four main triage principles: A) If the epidemiological history and presence of clinical symptoms are confirmed by the outpatient or emergency triage points, the patient shall be taken to the fever clinic by a designated person; B) Patients with an epidemiological history of COVID-19 shall be confirmed at the outpatient or emergency triage points and brought to the emergency for investigation and screening; C) Patients aged <50 years, without epidemiological history, and presenting only with fever (frontal/axillary temperature ≥37.3°C, ear temperature ≥37.5°C) should be triaged to the fever consultation room in the emergency department; D) If any hospital visitor other than the patient meets any of the epidemiological history criteria and presents clinical symptoms, the outpatient, emergency department, and inpatient entrance personnel shall directly inform the designated person to guide them to the fever clinic. If the visitor reports an epidemiological history without clinical symptoms within 14 days, they should not be allowed to enter the hospital area. For those presenting with only clinical symptoms or abnormal body temperature, emergency department consultation is recommended. The medical affairs department of The University of Hong Kong–Shenzhen Hospital conducts a preliminary review of the list of patients suspected of COVID-19, which is evaluated by the respiratory medical department for adults and the pediatric department for children; the financial department handles the admission procedures for the patients. Patients suspected of COVID-19 should be sent to the designated area using a negative pressure ambulance and transferred to the isolation ward by means of a dedicated channel. Nucleic acid testing should be conducted at least twice with an interval of 24 h, patients. If the result of the molecular test is positive, the patient will be transferred to the designated hospital (Third People’s Hospital of Shenzhen). Patients with improved clinical manifestations (eg, fever, respiratory symptoms, and lung condition) and 2 consecutive negative test results may be transferred to an ordinary ward or discharged for isolation at home for 14 d according to an assessment conducted by the specialist panel (Figure 2).

#### Patients in the Observation Ward

The presence of any 2 of the following points from 1 to 3, or point 4, indicate the need for hospitalization for observation:

1. Epidemiological history;
2. Fever/respiratory symptoms/diarrhea/fatigue/muscle pain;
3. Chest computed tomography (CT) findings of pneumonia;
4. Other situations that need to be observed.

***Check-in***: The patient should be escorted by a designated person through a special channel and admitted to a single room in the isolation ward. No one is allowed to enter or leave the room without permission, and no one is allowed to accompany the patient. Communication with treating physicians is mainly conducted by telephone.

***Diagnosis and treatment***: According to the patient’s condition, oral medicine (eg, antipyretics and cough suppressants) may be administered as required. Two nucleic acid tests should be performed with a 24-h interval.

***Release from observation***: Following 2 negative nucleic acid tests, the treating physician shall decide whether to terminate the observation. Follow-up should be conducted in the fever clinic for further consultation, and the treating physician shall issue the relevant medical documents.

***Follow-up treatment***: After follow-up in the fever clinic, the patient may consult a specialist for treatment, continue medical observation at home or at a designated place, or be admitted to the hospital according to medical advice.

## Instrument and Equipment Management

### Detailed Documentation of Equipment

A file for all medical equipment in the ward (eg, air purifier, air sterilizer, ultraviolet lamp, etc.) should be prepared. One machine 1 code (QR code), with dedicated management. Details regarding instrument and equipment files, as well as the operation status, maintenance, and repair, etc., should be recorded in a timely manner to ensure the normal operation of medical devices and equipment during the COVID-19 pandemic.

### Use of Robots for Delivery in the Isolation Ward

**Figure 3.**
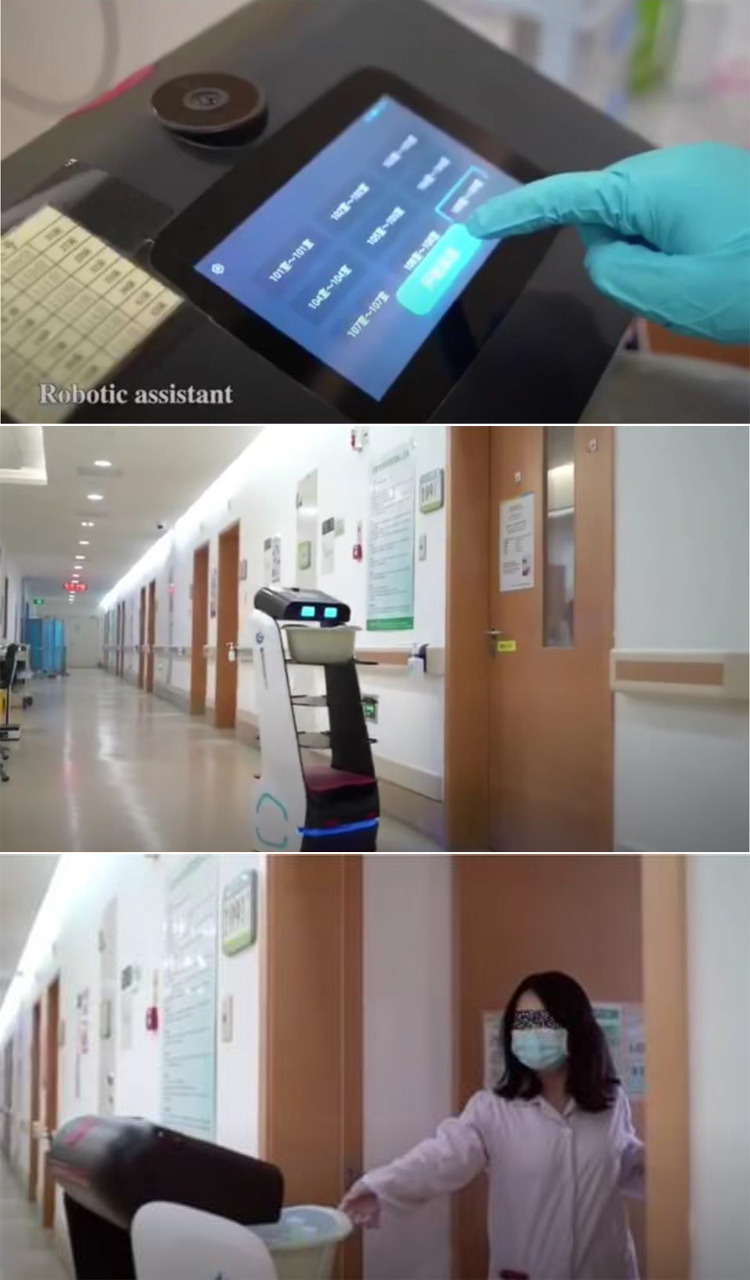
Robot for delivery in isolation ward.

As shown in Figure 3, robots can be used in the isolation ward to deliver medicines, daily supplies and meals, and reduce contact and the risk of cross-infection in a contactless manner. Robots can also function as a personal digital assistant scanning system and paperless office to minimize contact.

In ward management, long-term isolation can lead to depression and anxiety in patients. Therefore, we provided noncontact interactions to patients, such as emotional therapy of traditional Chinese medicine and the broadcasting of the 5-tone therapy tracks “Fresh vegetation,” “March in spring,” and “Return to the earth in spring” in “Huangdi’s Internal Classic.” This intervention exerts beneficial effects on patients, such as dredging of the liver and gallbladder, regulating *qi*, strengthening the spleen, and nourishing the blood. The 3 tracks should be played for 30 min twice daily (in the morning and evening).

## Logistics Management

### Application for Protective Equipment

The list of users, amounts of resources required for the following week, and registration record of protective equipment used in the previous week should be submitted to the medical consumables department as the basis for application.

### Distribution of Protective Equipment

All masks should be registered and issued to staff using their names. The high-risk departments (including emergency department, ICU, critical care unit, respiratory department, isolation ward, microbiology department, radiology department CT room) should prioritize the issuance of KF94 masks according to the actual needs. Surgical masks should be issued to non–high-risk clinical departments (standard: 2 masks per person per day for on-duty employees. Medical masks should be issued to administrative departments, property management personnel, and outsourcing personnel (standard: 1 mask per person per day) for on-duty employees.

## Cleaning, Disinfection, and Handling Procedures

### Establishment of Standards for Cleaning and Disinfection

The negative pressure isolation ward makes the air pressure in the ward lower than that in the surrounding adjacent communication area through air supply and exhaust equipment. The fresh air outside the ward is sent into the ward by the blower, and the air in the ward is discharged in an organized way after being treated, thus reducing the probability of cross infection of medical personnel and protecting the safety of the surrounding environment. The negative pressure isolation ward is used to isolate and treat patients with respiratory infectious diseases, and it is an important weapon to achieve victory against the epidemic. Therefore, the functions of the negative pressure isolation ward are mainly two-fold: First, through reasonable airflow organization, the air in the ward area flows directionally from the clean area to the polluted area, thus protecting the work safety of hospital medical workers. Second, indoor polluted air will be treated and discharged at the same time, which will not pollute the environment. The negative pressure isolation ward cools, dehumidifies or heats and humidifies the air in the process of air supply, so as to improve the comfort of indoor environment.

Traditional central air conditioners are prone to cross-infection due to the mixed use of indoor air in different areas. The negative pressure isolation ward adopts full direct current fresh air system, and the air supply system is set independently according to clean area and polluted area (including potential polluted area), so as to effectively prevent cross infection between medical staff and patients as well as patients in different wards.

**Figure 4.**
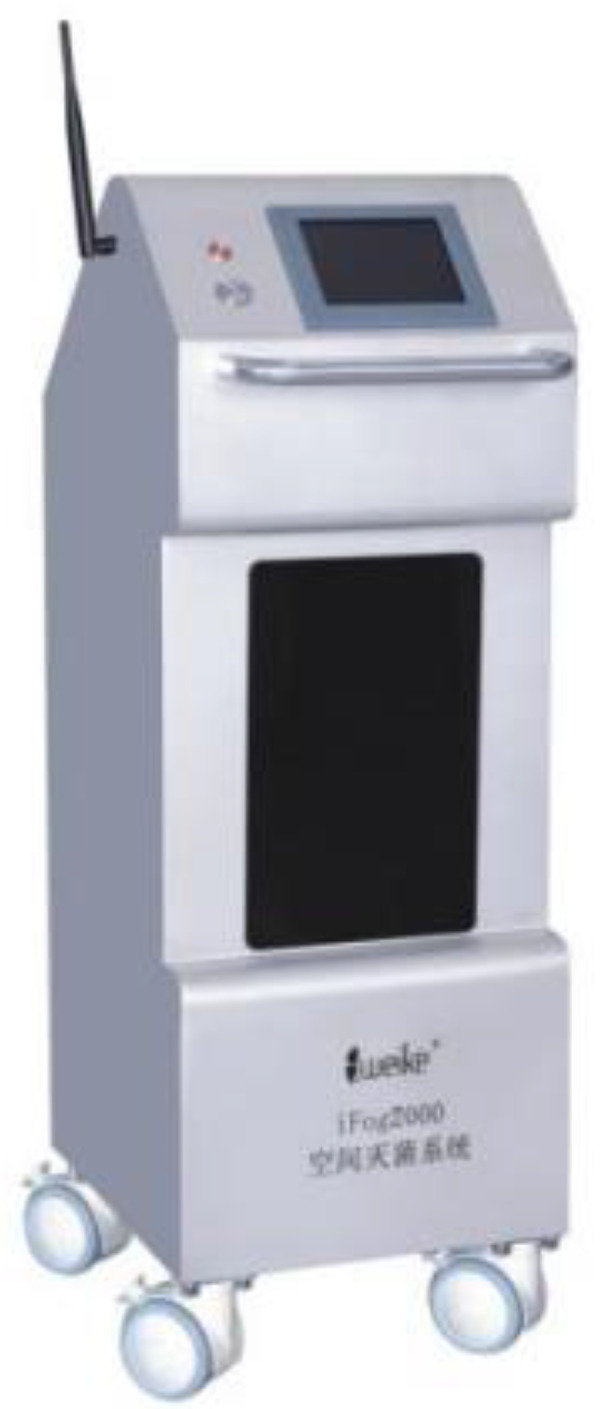
Atomized hydrogen peroxide machine.

In accordance with the “Hospital Environment Surface Clean and Disinfection Standard Operating Procedures”, the National Health Committee general office issued the “Medical Waste Management Guideline during the period of COVID-19 Outbreak”. A checklist of regional disinfection requirements was also produced, including location, air disinfection requirements and surface disinfection requirements (name of the disinfectant, method, concentration, duration, frequency). Air disinfection machines should be placed in the waiting hall of the fever clinic, screening clinic, and emergency temporary board room (waiting room when patients are in contact with other people). Atomized hydrogen peroxide (Figure 4) should be used for the terminal’s disinfection of positive patients and for weekly routine disinfection for areas where the suspected patient was examined or treated.

The air disinfection machine (model: kjf800; Sedley) runs continuously without affecting the work of the personnel. The hydrogen peroxide space sterilizer (model: ifog2000) is used for the disinfection of the terminals of patients with COVID-19; access during the disinfection process should not be permitted.

### Cleaning and Disinfection of the Isolation Ward

#### Clean Area (Office Area, Living Area)

Cleaning should be performed at least twice daily with 500 mg/L chlorine-containing disinfectant or disposable gamma wipes. Ultraviolet lamps should be used to disinfect the air daily for 60 min.

#### Inpatient Department

The patient rooms, nurse stations, corridors, and dirty cleaning rooms should be cleaned and disinfected with 1000 mg/L chlorine-containing disinfectant once daily. Bed units should be cleaned and disinfected from the outer to the inner area. The stethoscopes and axillary thermometers should be cleaned and wiped with 1000 mg/L chlorine-containing disinfectant. Also, 1000 mg/L chlorine-containing disinfectant should be used to wipe and disinfect high-frequency contact surfaces, such as keyboards, mice, inspection table surfaces, seats, and door handles at least twice daily. Computer keyboards and equipment with many gaps can be wrapped with plastic wrap, which should be replaced once or twice daily; however, this equipment should also be cleaned daily while replacing the plastic wrap. The 1000 mg/L chlorine-containing disinfectant should also be used for floor cleaning and disinfection. Blood, body fluids, and vomitus should be cleaned and disinfected with a gamma absorbent towel or 1000 mg/L chlorine-containing disinfectant. The 2000 mg/L chlorine-containing disinfectant is used to clean and disinfect toilets. The 1000 mg/L chlorine-containing disinfectant should also be used for the disinfection of terminals after discharge of patients with a negative nucleic acid test. Atomized hydrogen peroxide should be used for the disinfection of terminals after discharge of patients with a positive nucleic acid test. The sinks and floor drains of empty rooms should be irrigated at least once daily.

#### Cleaning and Disinfection of Patients’ Items

The daily necessities for patients should be provided by the hospital in principle. Personal belongings that are no longer needed can be disposed as infectious waste. The patient’s mobile phone and other personal belongings, as well as the outside of the duffel bag should be wiped with 75% alcohol at the time of discharge and returned to the patient.

#### Cleaning and Disinfection of Medical Records

Following discharge from the hospital, the medical record of the patient should be placed into a white transparent waterproof bag, and subsequently stored into a sealed box; high-level disinfection (ultraviolet light) should be performed twice weekly.

#### Disposable Protective Equipment

Disposable items, such as gowns, gloves, and caps, should be discarded as infectious waste. If the protective face shield is contaminated by blood or body fluids, it should be discarded as infectious waste.

#### Cleaning and Disinfection of Contaminated Clothing

The contaminated bedding and clothing should be collected in a white waterproof plastic bag marked “highly infectious fabric.” Before leaving the contaminated area, a layer of medical waste packaging bag should be added. The bags should be collected by the recycling personnel and sent to the washing center for cleaning and disinfection. The bedding of patients with a positive nucleic acid test should be disinfected and replaced immediately after transfer of the patient to other departments or discharge from the hospital. The curtains should be replaced; the used curtains should be put into a transparent bag, packed immediately, and sent for cleaning and disinfection. The bag should be marked “highly infectious fabric” after sealing. If wearing full protective coveralls, the replaced clothes should be recycled as normal work clothes.

#### Disposal of Medical Waste

All waste (including household waste, slippers, etc.) generated by patients suspected of COVID-19 should be treated as infectious waste. The infectious waste bucket should be foot-mounted. Waste from the isolation ward should be packed in double-sided yellow infectious waste bags (3/4 full), sealed, marked as “highly infectious clinical waste,” and sent for appropriate disposal. The labels on sharps containers should also indicate “highly infectious clinical waste.” If the external surface of the clinical waste and sharps containers is contaminated with infectious waste, an additional layer of packaging should be added. The contaminated area should be covered with a clinical waste packaging bag.

Toilet seat should be covered before flushing. If the patient has a drainage tube (urine tube, abdominal drainage tube, etc.), the drainage fluid can be poured gently into the toilet in the ward. After the drainage tube is removed, it can be placed into the infectious waste cask directly in the ward.

### Cleaning Supplies

Environment is cleaned with a variety of towels (1 towel per item). Dirty and clean areas are cleaned using different towels; towels of different colors are used for different areas (ie, white and red towels are used for the wards and hospital rooms, respectively). The management company should be responsible for cleaning, disinfecting, and drying the sanitary utensils after use. It is prohibited to clean and dry the towels, floor towels, and work clothes in the ward.

### Invasive Puncture

For invasive puncture (eg, peripherally inserted central venous catheter, central venous catheter, and extracorporeal membrane oxygenation), the skin surface should be disinfected with 0.5% iodophor.

### Comprehensive Psychological Nursing

During the pandemic, specialists should be available to provide psychological counseling to patients and their families, as well as to doctors and nurses. Psychological counseling is mainly provided through interaction by means of telephone, WeChat, email, and other network channels. In the isolation ward, soft music can be broadcasted to relieve the nervousness and anxiety of patients.

## Environmental Layout

### Regional Management of Infection Control

The number of entrances and exits of the outpatient building should be reduced to 3 entrances. Stations should be set up to monitor the temperature of individuals entering and exiting the clinic in combination with infrared temperature monitoring and thermal imaging.

### Special Transport Route for Patients Suspected of COVID-19

The person in charge of the elevator should be informed to shut down the elevator before the transfer. The security personnel and the disinfection personnel should be informed to take their positions before and after the transfer.

#### CT Examination

Patients suspected of COVID-19 should be transferred to the designated CT room by means of a special route. The route should be cleared before departure and the passageway should be disinfected after return.

#### Transfer to the Isolation Ward

Patients who can walk should wear a blue gown, surgical masks, and shoe covers. Air disinfection should be carried out in the isolation room after the transportation of patients in wheelchairs. After departure and return, the passageway, corridor, and door of the patient room should be cleaned and disinfected.

#### Transfer to the Designated Infectious Disease Hospital

If the nucleic acid test of a patient is positive, the physician should contact the medical affairs department. The medical affairs department shall contact the designated hospital of infectious diseases and arrange the ambulance service to transfer the patient. Once the ambulance arrives, the designated person should clear the passageway and control the elevator. Patients who are able to walk should wear a blue gown, NP5 mask, and shoe covers before leaving. For patients transported using a wheelchair, the wheelchair should be put into the isolation room for air disinfection.

## Personnel Training and Assessment

### Establishment of Infection Protection Standards for Medical Staff

To strengthen orientation, the hospital infection management department should conduct training on guidelines for the prevention and control of COVID-19, interpret the difficulties and countermeasures of hospital infection control,^2^ and answer queries regarding protective equipment, hand hygiene, and mask selection in the protection process.^3^

### Diverse Mandatory Training

To enhance the knowledge of medical personnel on COVID-19-related diagnosis, treatment, and guidelines, the diagnosis and treatment procedures should be standardized, and their awareness should be raised. Medical personnel focus on the investigation of the epidemiological history of patients with fever; detailed epidemiological history data can provide a reliable basis for the differential diagnosis of COVID-19.^4^ The University of Hong Kong–Shenzhen hospital has carried out COVID-19 prevention and control training in various forms and on multiple occasions, including self-protection against COVID-19, diagnosis and treatment, etc., in the form of limited on-site training, online training, real-time online Q&A, etc. This approach was used to reduce the contact of medical staff and eliminate their risk of infection. An online information exchange platform was established to timely understand and collect the latest news of the epidemic and provide updated information on prevention and control requirements at all levels, as well as targeted guidance on prevention and control measures. Other institutions should follow this practice.

### Supervision, Inspection, and Evaluation

The hospital should formulate specific implementation plans for supervision, inspection, and evaluation, carry out regular or irregular spot checks, and ensure the quality of the epidemic prevention and control measures through on-site inspection, interviews, questionnaire surveys, and assessment by means of self-assessment, mutual assessment, and third-party assessment.

Supervision, inspection, and evaluation mainly include:

(1) Organization and management, clear division of responsibilities and conscientious performance of duties; (2) Implementation of rules and regulations, including 3-level prevention and control, hospital infection control, personal protection, medical waste management, and disinfection and isolation; (3) Checking of the epidemic training, training plan, training content, training implementation, participation rate, and training effect; (4) Coordination and joint prevention and control with municipal designated infectious disease hospitals.

Based on the above self-evaluation and supervision, the situation should be analyzed and summarized, aiming for continuous improvement; timely feedback should be provided for any identified problems.

**Figure 5.**
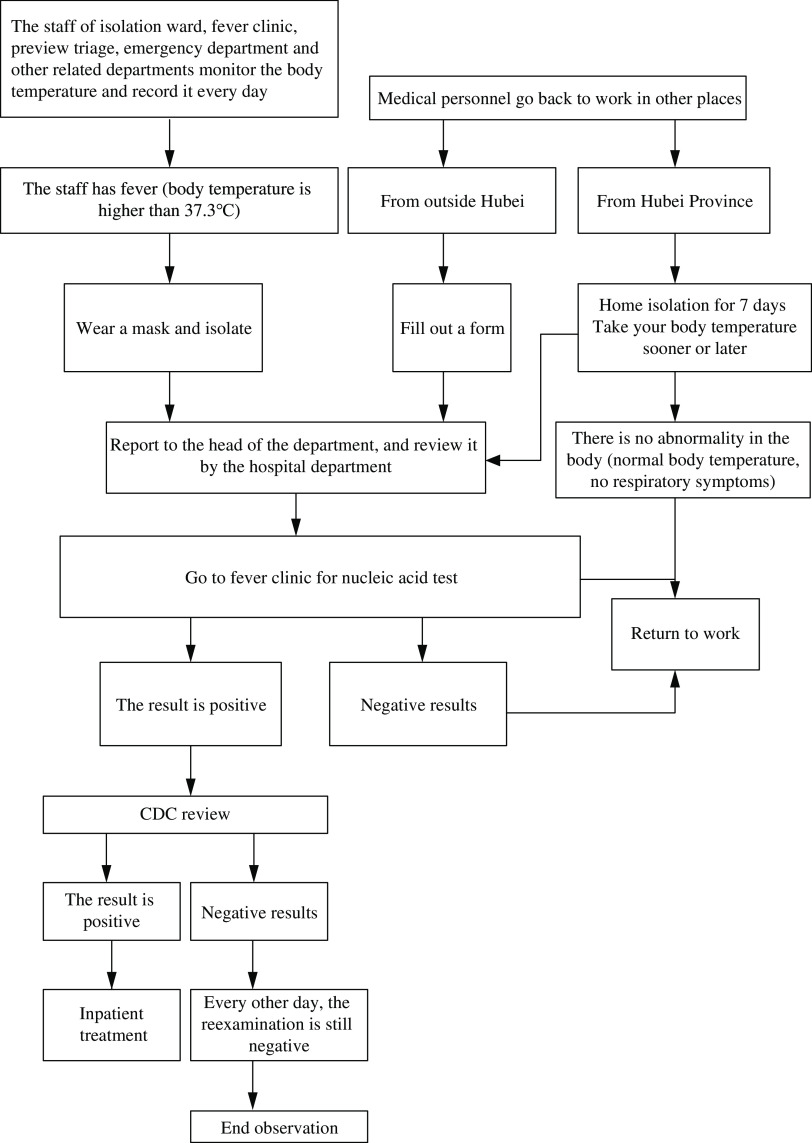
Health observation, report, and screening process applied by staff in isolated areas managing patients with coronavirus 2019 (COVID-19).

The health observation, report, and screening process applied by staff in isolated areas managing patients with COVID-19 are shown in Figure 5.

### Treatment Process in Response to Occupational Exposure of Medical Personnel to COVID-19

**Figure 6.**
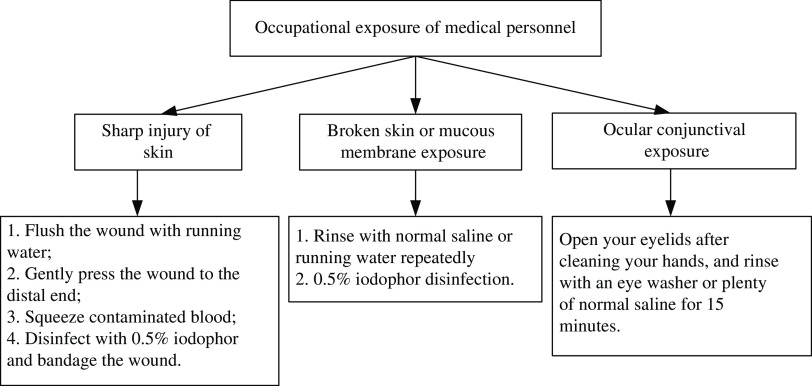
First step emergency management of occupational exposure.

Following occupational exposure to COVID-19, the incident should be reported to the department director and head nurse (Figure 6). The hospital department should be notified by telephone.

The Occupational Exposure Registration Form in the “Exploring the No.1 Hospital Infection Real-time Monitoring and Management System” on the hospital intranet should be printed and filled in; the department head should sign the form for confirmation.

Exposed individuals should fill in the Registration Form of Close Contact and the Observation Form of Close Contacts, and send the electronic versions to the hospital Infection Control Department; subsequently, these forms should be forwarded to the Centers for Disease Control (CDC).

The Department of Sensology will conduct health education and psychological assessment of the exposed individuals by telephone; the exposed individuals must complete the Post-traumatic Stress Disorder Checklist and sign the informed consent form for novel coronavirus prophylaxis drugs.

The exposed individual should be isolated on the day of exposure for 14 d.

If the exposure source is a patient with hepatitis B, syphilis, hepatitis C, human immunodeficiency virus (HIV), and other diseases, the incident should be handled according to the occupational exposure process of medical personnel.

The department of sensation of the hospital should provide the name and department of the exposed individual to the Western pharmacy by telephone. The department should assign other personnel to the outpatient Western pharmacy to collect the drugs and administer them within 2 h after exposure.

The department should assign other personnel to the hospital to submit the Occupational Exposure Registration Form, Post-traumatic Stress Disorder Checklist, and Informed Consent of Occupational Exposure Medication for filing and review.

Sensory staff of the hospital should issue the Notice of Occupational Exposure Inspection and Notice of Occupational Exposure Medication. The staff of the department should return the Notice of Occupational Exposure Medication to the Western pharmacy.

Following entry of the exposed individual to the designated isolation area, the municipal CDC is responsible for sampling. Individuals who develop any symptoms, (eg, fever, fatigue, dry cough, stuffy nose, runny nose, diarrhea) should promptly seek medical advice.

After 14 d of isolation, the exposed individual shall collect corresponding specimens for inspection according to the exposure process. Following a negative test, the exposed individuals return to their posts, and the Notice of Dismissal of Isolation is issued by the municipal CDC.

If the exposure sources are patients with hepatitis B, syphilis, hepatitis C, HIV, and other diseases, follow-up should be performed regularly according to the relevant regulations concerning occupational exposure.

## Results

From January 2, 2020, to April 23, 2020, The University of Hong Kong–Shenzhen Hospital received 198,802 patients. Of those, 10,821 were hospitalized; 26,767 were received by the emergency department and fever clinics; 288 patients were admitted for observation with fever; and 324 were admitted as suspected cases for isolation. All patients underwent effective screening and treatment; there was no report of hospital-acquired infection. The anti-epidemic work has been fully recognized by the authorities and all sectors of society. According to the data, patients with fever accounted for 0.152% of the cases (including those in the outpatient and emergency departments); patients suspected of COVID-19 accounted for 0.096% of the cases (including those in the outpatient and emergency departments), and confirmed cases accounted for 0.515% of patients with fever.

## Conclusions

Medical staff play a vital role in the prevention and control of COVID-19 transmission. Reasonable and feasible processes need to be developed for all health-care personnel in all departments during this critical period.

A team of experts on respiratory diseases, infection, infection control, imaging, emergency department, and clinical laboratory should be set up to accurately judge the conditions of patients in the emergency department, fever clinic, inpatient department, and isolation ward, and provide professional advice. Decisive measures should be taken to ensure that the medical work is carried out in an orderly manner. The present comprehensive response model may be useful in large public health emergencies to ensure appropriate management and protect the health and life of individuals.

## References

[1] Yu J, Hu L, Guo Q, et al. Practice of prevention and control strategy in outpatient of general hospital during COVID-19 epidemic. Chongq Med. 2020;49(15):2459-2462. http://kns.cnki.net/kcms/detail/50.1097.r.20200212.0819.004.html.

[2] Yao H, Suo J, Du M, et al. Difficulties and countermeasures in the prevention and control of nosocomial infection during the COVID-19 epidemic. Chin J Nosocomial Infect. 30(6):806-810.

[3] Li L, Wu A. Common confusion of hospital infection prevention and control in COVID-19. Chin J Infect Control. 2020;19(02):105-108. http://kns.cnki.net/kcms/detail/43.1390.R.20200209.1237.002.html. Accessed August 17, 2021.

[4] General Office of the National Health Commission. Notice of the General Office of the National Health Commission on strengthening the management of fever clinic and hospital infection prevention and control in key hospitals of key areas: medical letter of the National Health Office [2020] no. 102 [A]. 2020-02-03.

